# A service evaluation study of the impact of ageless policy in a London inner-city early intervention in psychosis service

**DOI:** 10.1192/bjb.2024.33

**Published:** 2025-04

**Authors:** Charlotte Johnston-Webber, Shreeya Gyawali, Elvan U. Akyuz, Madalina Zlate, Georgios Nerantzis, Nikita Beauvillain, Olivier Andlauer, Susham Gupta

**Affiliations:** 1 London School of Economics and Political Science, UK; 2 North East London NHS Foundation Trust, UK; 3 Avon and Wiltshire Mental Health Partnership NHS Trust, Bath, UK; 4 East London NHS Foundation Trust, UK; 5 Edouard Herriot Hospital, Lyon, France; 6 Barts and the London School of Medicine and Dentistry, Queen Mary, University of London, UK

**Keywords:** Early intervention for psychosis, psychotic disorders/schizophrenia, clinical outcomes measures, health economics, cost-effectiveness

## Abstract

**Background:**

Schizophreniform disorders tend to have an early onset. Early intervention in psychosis (EIP) services aim to provide early treatment, reduce long-term morbidity and improve social functioning. In 2016, changes to mental health policy in England mandated that the primarily youth-focused model should be extended to an ageless one, to prevent ageism; however, this was without strong research evidence.

**Aims and method:**

An inner-city London EIP service compared sociodemographic and clinical factors between the under-35 years and over-35 years caseload cohorts utilising the EIP package following the implementation of the ageless policy.

**Results:**

Both groups received similar care, despite the younger group having significantly more clinical morbidity and needs.

**Clinical implications:**

Our results may indicate that service provisions are being driven by policy rather than clinical needs, potentially diverting resources from younger patients. These findings have important implications for future provision of EIP services and would benefit from further exploration.

Early intervention in psychosis (EIP) is a subspecialty in psychiatry offering intensive treatment and support to those who are presenting with the first episode of a primary psychotic disorder such as schizophrenia (FEP). EIP services were first set up in the UK following their inclusion in the 1999 National Service Framework for Mental Health^1^ and are now the recommended standard of care.

Traditionally, EIP services were youth-focused and accepted referrals for individuals up to the age of 35 years. Schizophrenia has its greatest peak incidence in early adulthood,^2^ with much smaller peaks in middle age (40s) and again after age 60 (late-onset schizophrenia).^3^ Research also suggests that a longer duration of untreated psychosis (DUP) affects treatment response, resulting in poorer prognosis in terms of both clinical and social outcomes.^4^ EIP aims to reduce the DUP by offering quick assessment and rapid access to comprehensive mental health treatment and support. The EIP package includes medical care combined with care coordination, individual psychology (e.g. cognitive–behavioural therapy for psychosis), family intervention, peer support, education and employment support.^5^

Evidence has consistently demonstrated that facilitating early intervention and providing intensive treatment and support to young people presenting with FEP can greatly improve long-term outcomes in terms of education, employment and independent living.^6,7^ In addition, an economic analysis conducted in partnership with the London School of Economics demonstrated that although properly resourced EIP teams required substantial initial investment, there were significant long-term clinical and cost benefits of preventive strategies.^8^ These savings were realised mainly through reduced formal admissions and improved employment.

The 2014 National Institute of Health and Care Excellence guidelines stated: ‘Early intervention in psychosis services should be accessible to all people with a first episode or first presentation of psychosis, irrespective of the person's age or the duration of untreated psychosis.’^5^ As a result, it was mandated that all EIP services in England should be ‘ageless’ and accept referrals for anyone presenting for the first time with psychotic symptoms, regardless of age.^5^

The clinical presentation and needs of patients aged over 35 years are likely to differ from those under 35. A prolonged DUP in patients with previously undetected and chronic psychosis, a higher prevalence of disorders such as affective psychosis and persistent delusional disorders, and greater physical health comorbidities are all more likely in older patients.^3,9–11^

Despite the expansion of EIP services to the over-35 group in September of 2016, there have been few studies examining the differences in characteristics and needs of these two age groups or attempting to evaluate the impact of the change in policy.^9–12^ This service evaluation of an inner-city London EIP service aimed to examine a wide range of demographic and service use data for a group of 50 patients over 35 years old and another 50 under 35 years old. The main aim of the study was to explore the impact of the ageless policy on care and service delivery by comparing the demographic and clinical profile of the two groups with respect to their utilisation of the early intervention package and allied clinical services.

## Method

### Study setting

The study data were collected from the City and Hackney Early Intervention Service (EQUIP), which is part of the East London NHS Foundation Trust. The London Borough of Hackney and the City of London have a combined population of just under 300 000.^13^ The population is relatively young and highly mixed from socioeconomic, ethnic and cultural perspectives.^13^ The vast majority of referrals to the EQUIP team are from the more populous Borough of Hackney. City and Hackney is ranked highest in terms of incidence of new cases of psychosis in England.^14^ The team takes referrals of patients between the ages of 18 to 65 years, as well as graduates from child and adolescent mental health services. Patients with first-episode or known presentation of psychosis needing hospital admission are managed by a specialist EIP in-patient arm. Exclusion criteria for acceptance under EIP include organic psychosis, drug-induced psychosis and primary trauma-related disorders. EQUIP receives referrals from primary, secondary and other statutory services but does not accept self-referral. Patients have access to medical care, care coordination, psychology, family therapy, education and employment support, occupational therapy and physical healthcare within a multidisciplinary integrated team. The team can offer assertive outreach care and has access to crisis resolution and home treatment team (HTT) services and substance misuse services. Patients accepted to the team can remain under the service for up to 3 years based on clinical need. Patients can be discharged back to their general practitioner or referred for further specialist mental healthcare support, dependent on diagnosis, complexity, level of recovery and clinical needs.

### Data collection

Consecutive referrals from September 2016 consisting of a sample of 50 patients under 35 years old and 50 over 35 years old were included in the study. This was done for convenience and to minimise selection bias. Individuals under the care of the team for less than 6 months from the date of referral were excluded. Three individuals fell into this category, and the next three cases were chosen from the referral list to make up the sample size. Anonymised data were collected from electronic patient records (RiO). Data were stored as per local trust and National Health Service data protection guidelines in accordance with the clinical governance policy. Raw data were not shared with any third party. The study was meant to be descriptive, and few statistical analyses of the data were carried out owing to the relatively small sample size. Where deemed useful, *P*-values were calculated by two-tailed t-tests using the statistical R software (version 4.1.1).

The following anonymised data were collected for each individual as recorded on RiO.

- Demographic parameters – these included age at referral, gender, ethnicity, marital status, employment status, presence of dependent children, housing situation.
- Clinical parameters – these included source of referral, ICD-10 diagnosis, DUP and any recorded mental or physical health and substance use comorbidities.
- Treatment and service use parameters – these included treatment with antipsychotic medication, number of clinical contacts including care coordinator contacts, medical contacts, number of family and/or individual psychology sessions, and occupational therapy and support worker contacts, as well as time to discharge. Number of hospital admissions, HTT episodes and detentions under the Mental Health Act (MHA) while under EQUIP were also collected. Only subsequent hospital admissions and HTT episodes were counted once a referral had been accepted by EQUIP. Initial referrals from in-patient units or HTT were not counted. Only discrete HTT episodes (not associated with in-patient admissions) were included when seen as an alternative to hospital admission.

### Ethical statement

Local permission to conduct this study was granted by the Clinical Governance and Ethics Committee of the East London NHS Foundation Trust. The project was also approved by the London School of Hygiene and Tropical Medicine MSc Research Ethics Committee (reference number 16881).

## Results

### Demographic parameters

The mean age at time of referral in the patients aged under 35 years was 23.8 years, and the median was 22 years. The mean age in the over-35 group was 46.3 years, and the median was 45 years. There were significant differences in ethnic distribution between the two groups. Non-White groups formed 66% of the under-35 patients, compared to 38% of the over-35 group. White groups represented 58% of the over-35 group. Over-35s were more likely to have dependent children and to have children's social care involvement than their under-35 counterparts (*P* = 0.004). The younger group were much more likely to be in education, and there were no significant differences across the two groups in terms of employment. The majority of the under-35s were single (86%), compared with 40% of the over-35s, although the over-35s were more likely to be living alone than the under-35s (38% *v.* 20%). The demographic details of individuals in the study are given in Table 1.

**Table 1:** Demographic parameters of individuals at point of referral^a^

		Under 35 (%) *n* = 50	Over 35 (%) *n* = 50	Significance
Age, years	Mean (s.d.)	23.8	46.3	
	Median (IQR)	22	45	
Gender	Male	25 (50)	27 (54)	NS
	Female	25 (50)	23 (46)	
Ethnicity	Black, Asian and mixed	33 (66)	19 (38)	*P* < 0.05
	White and White other	17 (34)	29 (58)	
	Other/not recorded	0 (0)	2 (4)	
Dependent children	Yes	8 (16)	21 (42)	*P* < 0.05
	No	42 (84)	29 (58)	
	Children's social care involvement	5 (10)	10 (20)	
Employment status	Yes	15 (30)	15 (30)	NS
	No	35 (70)	35 (70)	
Housing situation	Alone	10 (20)	19 (38)	NS
	With family	28 (56)	24 (48)	
	Shared	8 (16)	5 (10)	
	Temporary	2 (4)	2 (4)	
	Homeless	2 (4)	0 (0)	
Marital status	Single	43 (86)	20 (40)	NS
	Married/partner	5 (10)	13 (26)	
	Separated/divorced	2 (4)	14 (28)	
	Widowed	0 (0)	3 (6)	

NS, not statistically significant.

a. Results are given as *n* (%) unless otherwise specified, and all information is at point of referral.

### Clinical parameters

In our view, the small sample size did not allow very meaningful comparison of the clinical presentations, especially given the heterogeneity of the psychotic presentations. The findings are described in Table 2 for context.

**Table 2:** Clinical parameters of individuals in current study

		Under 35 (%) *n* = 50	Over 35 (%) *n* = 50
Referral source	Single point of access	15 (30)	16 (32)
	In-patient units	23 (46)	18 (36)
	Home treatment and crisis resolution team	0 (0)	7 (14)
	EIP transfer	2 (4)	2 (4)
	Primary care liaison	2 (4)	4 (8)
	Community mental health team	0 (0)	1 (2)
	Child and adolescent mental health service	5 (10)	0 (0)
	Specialist personality disorder service	0 (0)	2 (4)
	ARMS	2 (4)	0 (0)
	Her Majesty's Prison	1 (2)	0 (0)
Primary diagnosis	Non-affective psychosis	43 (86)	35 (70)
	Manic psychosis	2 (4)	2 (4)
	Depressive psychosis	2 (4)	8 (16)
	Persistent delusional disorder	0 (0)	2 (4)
	Postnatal psychosis	1 (2)	1 (2)
	Post-traumatic stress disorder	1 (2)	1 (2)
	Drug-induced psychosis^a^	1 (2)	0 (0)
	Emotionally unstable personality disorder	0 (0)	1 (2)
Significant physical health comorbidity	Yes	9 (18)	17 (34)
	No	41 (82)	33 (66)
Significant mental health comorbidity	Yes	16 (32)	21 (42)
	No	34 (68)	29 (58)
Smoker	Yes	13 (26)	19 (38)
	No	37 (74)	31 (62)
Harmful alcohol use	Yes	3 (6)	10 (20)
	No	47 (94)	40 (80)
Cannabis use	Yes	16 (32)	7 (14)
	No	34 (68)	43 (86)
Other illicit substance use	Yes	3 (6)	6 (12)
	No	47 (94)	44 (88)

ARMS, At Risk Mental State service (an early detection service run by the EQUIP team staff).

a. Patient was diagnosed with a non-organic psychosis, but initial diagnosis for drug-induced psychosis had not been altered in records.

### Duration of untreated psychosis

DUP is a concept in primary schizophreniform disorder. This cohort includes a wide range of psychotic presentations including unipolar and bipolar affective disorders, other psychiatric complexities and dual diagnoses (similar to most EIP services in the UK). This made it difficult to draw any significant inferences. The differences between the groups were not statistically significant (NS), but they showed a likelihood of more acute presentation in the under-35s.

#### EIP package and community service use

The duration of care under the EIP team was 20.86 months in the under-35s and 17.75 months in the over-35s.

There were a total of 2289 clinical contacts for the under-35s, and 1973 contacts for the over-35 group (NS) during these periods. The average number of contacts for the under-35s was 1.98 per month, compared with 1.87 contacts per month for the over-35s (NS). The average number of care coordinator contacts per month was 1.09 in the younger group, and 0.98 in the older group (NS). The under-35s had an average of 0.34 medical contacts per month, and the over-35s had 0.39 (NS). The average number of total psychology contacts per month was 0.44 for the under-35s and 0.41 for the over-35s (NS).

Psychology data showed that 72% (*n* = 36) of under-35s and 60% (*n* = 30) of over-35s had received psychology input. Sixteen per cent (*n* = 8) of under-35s had family and individual therapy, as compared with 6% (*n* = 3) over 35s who had both family and individual therapy combined. Forty-eight per cent (*n* = 24) of under-35s had individual therapy, compared with 50% (*n* = 25) of over-35s. Eight per cent (*n* = 4) had only family therapy in the under-35 group, compared with 4% (*n* = 2) of over-35s. The differences in contacts with support workers and occupational therapists were also not clinically significant. These are described in Table 3.

**Table 3:** Total contacts, contacts by professional type and average contacts per month

Contact type	Under 35 (*n* = 50)	Over 35 (*n* = 50)
Care coordinator	1265	1038
Medical	396	407
Individual psychology	413	384
Family therapy	91	49
Occupational therapist	66	53
Support worker	58	42
Total	2289	1973
Cumulative months of data	1157	1057
Average total contacts per month	1.98	1.87
Average care coordinator contacts per month	1.09	0.98
Average medical contacts per month	0.342	0.385
Average total psychology contacts per month	0.436	0.410

#### In-patient care and use of crisis pathway under EIP service

In the under-35 group, there were a total of 32 in-patient admissions, compared with 12 admissions in the over-35 group. Twenty individuals (40%) in the under-35 group and eight individuals (16%) in the over-35 group were admitted to an in-patient unit (*P* = 0.008). The total numbers of detentions under the MHA were 23 in the under-35 group and 9 for the over-35s. Fifteen individuals in the under-35 group (30%) and six individuals in the over-35 group (12%) were detained under the MHA (*P* = 0.027). The numbers of HTT admissions were 8 in the under-35 group and 2 in the over-35 group. Along with more in-patient admissions and crisis pathway care and higher rates of detention, the younger group were more likely to experience readmission and be on depot antipsychotic medication and clozapine therapy for treatment-refractory schizophrenia (Table 4).

**Table 4:** Treatment and service use parameters of individuals in the current study

		Under 35 (%) *n* = 50	Over 35 (%) *n* = 50	Two-proportions *z*-test (*P*-value)
In-patient admissions	Number of individuals with admission to MH ward	20 (40)	8 (16)	0.008**
	Total number of admissions to MH ward^a^	32	12	
MHA detentions	Number of individuals who had a detention under the MHA	15 (30)	6 (12)	0.027**
	Total number of detentions under the MHA^a^	23	9	
HTT episodes	Number of individuals who had a HTT episode	7 (14)	2 (4)	0.081
	Total number of HTT episodes^a^	8	2	
Antipsychotic medication	Any antipsychotic medication	44 (88)	43 (86)	0.766
	Depot antipsychotic medication	8 (16)	1 (2)	0.014**
	Clozapine treatment	3 (6)	0 (0)	0.079
Duration under early intervention	Average time to discharge (in months)	20.86	17.75	

MH, mental health; MHA, Mental Health Act; HTT, home treatment team.

a. Some individuals had more than one episode of admission, detention or care under HTT.

** *P* < 0.05.

## Discussion

This is an initial evaluation of the potential impact of the age-inclusive policy in England on the utilisation of the clinical EIP care package. The study was conducted in an inner-city ethnically diverse urban population and in a team that routinely manages clinically complex cases and does not use narrow diagnostic criteria for psychosis. Our study found that the two groups, under 35 years old and over 35 years old, were similar with respect to many demographic parameters. However, the younger cohort were more likely to be single or living with their primary families and in education, whereas older patients were more likely to be living with their own families (often with dependent children) or to be separated or divorced. Black and other minority ethnic (BAME) patients were overrepresented in the under-35 group, with ‘White’ groups being overrepresented among the over-35s. The difference in ethnic distribution between the two groups is likely to be reflective of the local population. However, this distribution reveals a clear divergence in care needs in terms of family work, adult and child social care involvement, and educational support between the groups. Moreover, there is well-established evidence of higher rates of detention under the MHA, use of section 136 and community treatment orders in BAME groups compared with their White counterparts in England.^15^ In addition, those aged 18–34 years are most likely to be placed under a section 136 order and detained under the MHA.^15^ Despite these differences, the study found very similar uptake of the EIP care package between the two groups.

The size of the evaluation and the heterogeneous nature of the clinical presentations does not allow any meaningful conclusions around diagnoses and DUP. However, it is well-known that the rate of first presentation of schizophreniform and bipolar affective spectrum disorders is significantly higher in younger populations, whereas there is greater prevalence of depressive psychosis, chronic disorders, longer DUP and physical health comorbidities in older groups.^10,11^

Another area where the two groups differed significantly was that the under-35 group had more hospital admissions, detentions under the MHA, readmissions and HTT usage than the over-35 group. These differences in the service use between the two groups have been replicated by other studies.^12,16^ Although the majority of individuals in both groups had been prescribed antipsychotic medications, notably more of the younger patients had been prescribed long-acting depot injection, which tends to reflect poor compliance with treatment and associated high risk. In addition, the use of clozapine was higher in the under-35s. This study did not look at the criminal justice involvement and forensic input of the two groups, but anecdotally there are differences, especially when ethnic differences are taken into account.

The most interesting finding from this service evaluation was that despite the clear clinical and sociodemographic differences between the groups, there was no corresponding difference in the way the EIP care was being delivered to them by this team. This finding suggests that the care provided to the two groups was similar in most domains, despite the clear differences in clinical and social needs, raising questions around equity.

The probable explanation is that care is being delivered based on policy and service-driven factors, rather than on factors related to the clinical needs of the patients or actual research evidence. This also points towards diversion of resources from under-35s to over-35s, as well as potentially further widening the existing resource gap for BAME groups. Hence, a policy-driven effort to reduce ageism through extending the youth-focused model to a more generic one may be creating a reverse issue of resource diversion. There needs to be further exploration of the unintended consequences of funding of EIP services on generic community mental health teams, including the further expansion to ageless EIP services.

Finally, the delivery of the components and clinical outcomes of EIP packages in such services nationally are closely monitored through an annual National Clinical Audit for Psychosis (NCAP)^17,18^ that rates the ‘performance’ of EIP teams accordingly. We feel that focusing on NCAP may further divert the focus from patient-centric care to one excessively led by management and performance outcomes, which can then become the primary drivers of service delivery and resource distribution. We accept that this is not intentional but the unintended consequence of a top-down policy change as opposed to one based on good clinical and research-based evidence. In view of the significantly higher rates of readmission, detention under the MHA, HTT usage and use of depot medication in the under-35 group, we also propose that the merits of establishing an assertive outreach programme for under-35s in EIP should be considered.

The findings of this study must be interpreted with some caution. We studied a small sample of a ‘real-world’ cohort of patients with particular demographic make-up and significant clinical heterogeneity under one service in a diverse inner-city location. This was a pilot study, and the findings may not be generalisable to other locations or services but provide a good opportunity for reflection on the way care is currently being delivered by EIP services nationally. This should also alert other national health services that may be intending to follow the UK model of expansion to an ageless model. It also exposes the need for further clinical research on outcomes and a rethinking of the way these services are evaluated. The data were retrospectively collected and were of high quality as they were gathered from objective records from the electronic patient-record system. Efforts were made by researchers to reduce inaccuracies such as duplication of clinical contacts in both groups.

There are distinct advantages to proving intensive care for older patients with emerging mental health conditions; however, EIP services are intended to be youth-focused and aimed at management of schizophreniform disorders. In conclusion, this study showed important differences between individuals in the over- and under-35 groups under the care of the EQUIP team in City and Hackney. These differences related to both certain demographic characteristics – for example, living alone and having dependent children – and to certain service use features, in particular admissions to hospital and detentions under the MHA. Despite the limitations of the small study size and convenient sampling method, there is enough evidence to suggest that further studies are done to investigate the outcomes for different age groups under EIP services. At present, service delivery may be described as ‘one size fits all’, which may not be ideal. The result of this approach is that everyone accepted for the service is provided with the same level of clinical input irrespective of their individual needs, which does not address the issue of equity. Based on the findings of this study, we propose that delivery of EIP packages should be based on clinical needs, and that the blanket ‘ageless’ policy should be revisited with a view to planning services which are better suited to the differing needs of younger and older patients.

## Data Availability

The data that support the findings of this study are available on request from the corresponding author, S.G., for quality-check purposes only. The more granular and individual-level data are not provided publicly as they did not further influence the authors' conclusion.

## About the authors

**Charlotte Johnston-Webber**, MBChB, MSc, is a consultant psychiatrist and senior policy associate at the Department of Health Policy, London School of Economics and Political Science, London, UK. **Shreeya Gyawali**, MBBS, MD, MRcPsych, is a staff grade psychiatrist at North East London NHS Foundation Trust, London, UK. **Elvan U. Akyuz**, MD, MRCPsych, AFHEA, is a consultant psychiatrist at North East London NHS Foundation Trust, London, UK; **Madalina Zlate**, MBBS, MRCPsych, is a core trainee in psychiatry at Avon and Wiltshire Mental Health Partnership NHS Trust, Bath, UK. **Georgios Nerantzis**, MBBS, MRCPsych, is a consultant psychiatrist at East London NHS Foundation Trust, London, UK. **Nikita Beauvillain**, MD, is a consultant psychiatrist at Edouard Herriot Hospital, Hospices Civils de Lyon, Lyon Est Faculty of Medicine, Université Claude Bernard Lyon 1, Lyon, France. **Olivier Andlauer**, MD, is Honorary Senior Clinical Lecturer at Barts and the London School of Medicine and Dentistry, Queen Mary, University of London; and a consultant psychiatrist at EQUIP & Heads UP, City and Hackney, East London NHS Foundation Trust, London, UK. **Susham Gupta**, MBBS, MSc, FRCPsych, is a consultant psychiatrist at EQUIP & Heads UP, City and Hackney, East London NHS Foundation Trust, London, UK.
